# Supplementary material for: Design and Validation of a Process Based on Cationic Niosomes for Gene Delivery into Novel Urine-Derived Mesenchymal Stem Cells
Source: Pharmaceutics. 2021 May 11;13(5):696. doi: 10.3390/pharmaceutics13050696 (PMC8151286; doi:10.3390/pharmaceutics13050696)
Supplement: Supplementary file 1 [file pharmaceutics-13-00696-s001.zip › pharmaceutics-1184082-supplementary.pdf]

pharmaceutics

Supplementary Materials: Design and Validation of a Process Based on Cationic Niosomes for Gene Delivery into Novel Urine-Derived Mesenchymal Stem Cells

Yerai Vado, Gustavo Puras, Melania Rosique, Cesar Martin, Jose Luis Pedraz, Shifa Jebari-Benslaiman, Marian M. de Pancorbo, Jon Zarate and Guiomar Perez de Nanclares

1. Materials and Methods

1.1. Influence of Culture Medium

Genomic DNA was extracted from cells using the QIAamp DNA Mini Kit (QIAGEN, Hilden, Germany), following the manufacturer's instructions. Total RNA from the cells was obtained using the QIAamp RNA Blood Mini Kit (QIAGEN).

Dosage and methylation analyses for multilocus imprinting defects (MLIDs) were carried out using methylation-specific multiplex ligation-dependent probe amplification with the ME034-A1 kit (MRC-Holland, Amsterdam, the Netherlands), following the manufacturer's recommendations.

Agarose gel electrophoresis was carried out on a 1.5% (*w*/*v*) agarose gel in TAE buffer. DNA was analyzed by direct sequencing using the 3500 Series Genetic Analyzer.

1.2. Osteogenic Induction

Cells were seeded in triplicate at a density of 7.5×10^3 cells/cm^2 in 12-well culture plates and cultured for 2 days in expansion medium to approximately 80% confluence. Then, the medium was replaced by osteogenic differentiation medium.

The osteogenic differentiation of the cells was induced for 3 weeks using the StemPro® Osteogenesis Differentiation Kit (Gibco-ThermoFisher Scientific, Waltham, MA, USA), following the manufacturer's instructions. Negative controls were cultured in Dulbecco's modified Eagle's medium containing 2 mM L-glutamine (DMEM, low-glucose, Gibco-ThermoFisher Scientific) and supplemented with 10% FBS.

The medium was changed every 3–4 days for 21 days. After 3 weeks of culture in osteocyte medium, matrix mineralization was assessed by Alizarin Red staining for calcium deposits according to the following protocol: culture supernatants were removed and the cells were washed twice with 1× PBS. The cells were fixed in 4% paraformaldehyde for 30 min at room temperature, washed three times with distilled water, and incubated with 2% Alizarin Red S (Merck KGAa, Darmstadt, Germany) for 25 min at room temperature. Then, cells were washed once with PBS and twice with distilled water. Images were obtained using an inverted microscope (NIKON Eclipse TS 100, Nikon, Tokyo, Japan) and a color camera (model ICD-879P, Ikegami, Tokyo, Japan).

Relative calcium content was quantified by dissolving mineralized regions with 10% acetic acid (PanReac) for 30 min at room temperature. Each sample was transferred to a 1.5 mL microcentrifuge tube. After vortexing for 30 s, samples were heated at 85 °C for 10 min. Tubes were incubated on ice for 5 min. After centrifugation at 15,000 rpm for 20 min, the supernatants were transferred to a new tube, and 10% ammonium hydroxide (PanReac) was added to neutralize the acid. Absorbance was measured at 405 nm.

2. Results and Discussion

2.1. Influence of Culture Medium

Nucleic acid integrity at different passages was assessed by agarose gel electrophoresis (see Figure S1A), polymerase chain reaction (PCR) amplification, and Sanger sequencing (data not shown). The obtained genomic DNA and total RNA were of good

quality. Moreover, PCR and Sanger sequencing were performed on the DNA, and a sequence of good quality was obtained, leading to the conclusion that the DNA was good enough to work with. No alterations in terms of methylation levels in different imprinted loci or in genome dosage during passages were found (see Figure S1B).

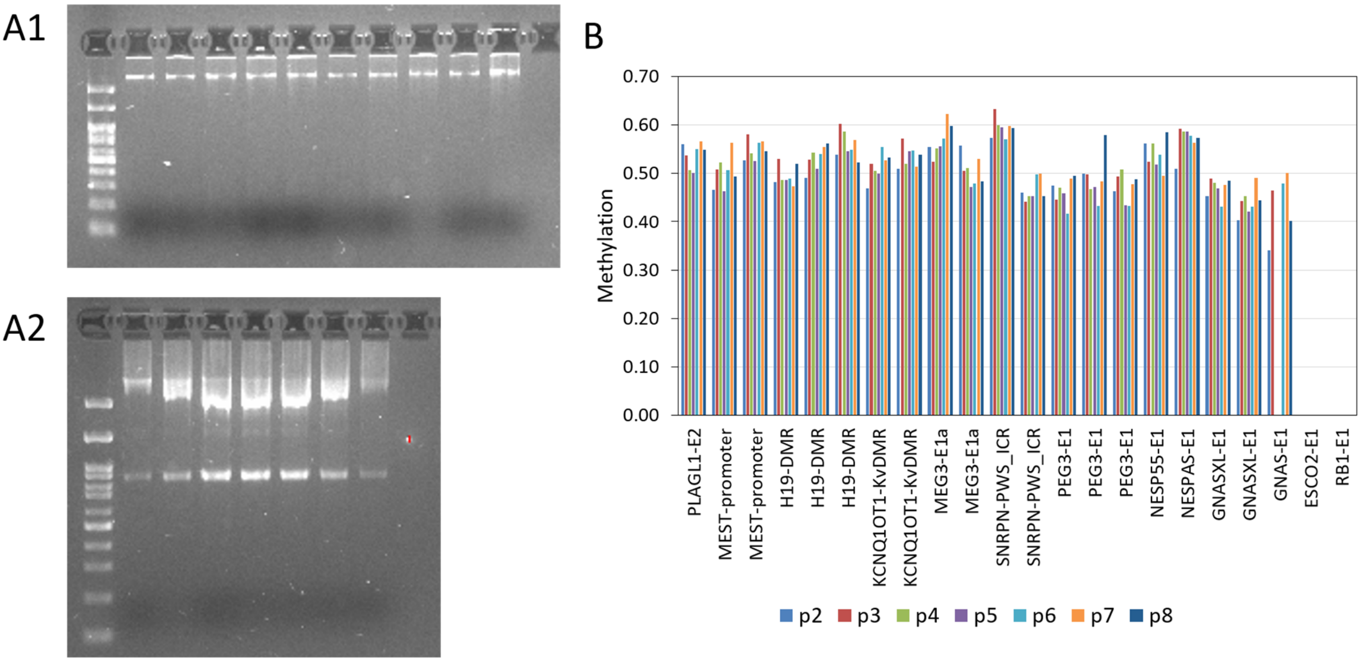

Figure S1. Nucleic acid integrity and methylation status stability of the cell culture. (**A**) Agarose gel electrophoresis of (A1) genomic DNA, where the bright band in the upper part of the gel demonstrates that the genomic DNA is not degraded, and (A2) total RNA of the cells, where the different bands correspond to the pattern expected for ribosomal RNA. (**B**) MS-MLPA study of multilocus imprinting disorders. Cells were tested at different passages for different imprinted loci.

2.3. Osteogenic Induction

Together with the presence of specific surface markers, as analyzed by flow cytometry, osteogenic differentiation is a characteristic that defines mesenchymal stem cells. After Alizarin Red staining, calcium accumulation became visible in cells treated with differentiation medium but not in negative controls (Figure S2A–D). Moreover, in addition to staining the samples, the calcium was quantified by measuring the absorbance at 405 nm (Figure S2E). In this quantification, every measurement of the differentiated cells showed statistically significant differences with the negative control. Considering all the above, it can be concluded that differentiation into osteocytes occurred in treated but not in control cells. Therefore, this was further evidence for classifying the cells isolated from urine as mesenchymal stem cells.

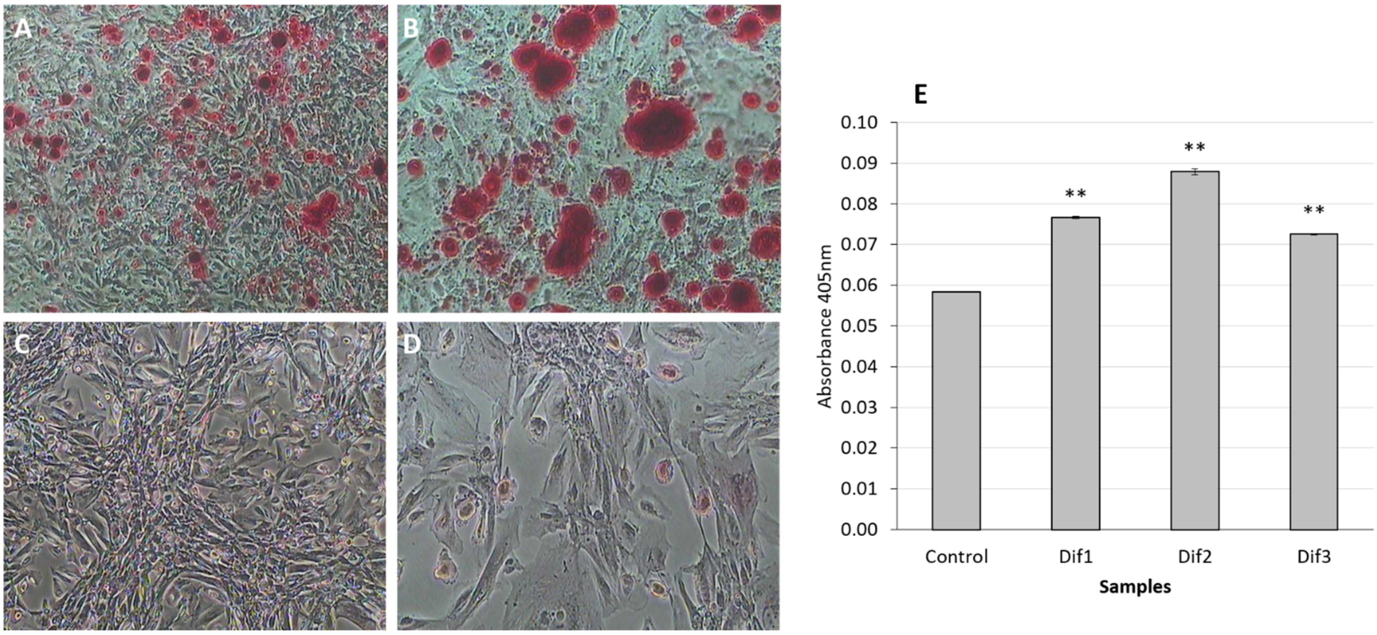

Figure S2. Alizarin Red-stained hUSCs. Reverse-phase microscope photographs for differentiated cells (A: 4×, B: 10×) and negative controls (C: 4×, D: 10×) are shown. Stained in red, calcium accumulations are observable in (**A**) and (**B**) but not in (C) and (D). (E) Osteogenic differentiation quantification. Absorbance data at 405 nm are presented. Mann–Whitney's statistical test was performed, and significant differences (** $p < 0.05$) were obtained between the control and each group of differentiated cells. Taken altogether, this confirms that treated cells differentiated into osteocytes, whereas negative controls did not.
